# Annotated checklist of the recent and extinct pythons (Serpentes, Pythonidae), with notes on nomenclature, taxonomy, and distribution

**DOI:** 10.3897/zookeys.66.683

**Published:** 2010-11-04

**Authors:** Wulf D. Schleip, Mark O’Shea

**Affiliations:** 1 Fichtenweg 11, 53340 Meckenheim, Germany; 2 Australian Venom Research Unit, Dept. Pharmacology, University of Melbourne, Vic., 3010, Australia; 3 Reptile Department, West Midland Safari Park, Bewdley, Worcs., DY12 1LF, United Kingdom

**Keywords:** snakes, ICZN, *Antaresia*, *Apodora*, *Aspidites*, *Bothrochilus*, *Broghammerus*, *Leiopython*, *Liasis*, *Morelia*, *Python*, taxa

## Abstract

McDiarmid et al. (1999) published the first part of their planned taxonomic catalog of the snakes of the world. Since then, several new python taxa have been described in both the scientific literature and non-peer-reviewed publications. This checklist evaluates the nomenclatural status of the names and discusses the taxonomic status of the new taxa, and aims to continue the work of McDiarmid et al. (1999) for the family Pythonidae, covering the period 1999 to 2010. Numerous new taxa are listed, and where appropriate recent synonymies are included and annotations are made. A checklist and a taxonomic identification key of valid taxa are provided.

## Introduction

Pythons (family Pythonidae) represent a family of non-venomous basal snakes within the superfamily Pythonoidea Fitzinger, 1826 (*sensu* Vidal et al. 2007, Vidal and Hedges 2009). Although present in Europe during the Miocene, and probably since the late Eocene (Szyndlar and Rage 2003), pythons are now restricted to the warmer regions of the Old World, ranging from Africa through South and Southeast Asia, Indo-Malaysia and New Guinea, to Australia (Kluge 1993, Scanlon 2001, Rawlings and Donnellan 2003, Rawlings et al. 2008). More than two thirds of the currently recognized extant species are found in the Australo-Papuan region (Kluge 1993, Scanlon 2001, Rawlings et al. 2008), where they have the greatest level of morphological and genetic diversity (Heads 2002, Rawlings and Donnellan 2003), and a high degree of endemism (Harvey et al. 2000, Rawlings et al. 2004). Whereas two Asian species (Python molurus and Python bivittatus) range north of the Tropic of Cancer, an African and at least seven Australian species extend their ranges south of the Tropic of Capricorn. Pythons occur in a variety of habitats, from desert and savanna, to subtropical and tropical rainforest (Kluge 1993) and into seasonally flooded grasslands and paddifields. Most species are terrestrial, some are arboreal (Kluge 1993) and a few are semi-aquatic. The 40 recognized extant species range in maximum adult length from 0.61 m to 10.0 m, and include the longest extant snake species.

### Taxonomic changes since 1999

McDiarmid et al. (1999) has become a standard reference for snake taxonomists. Since then python systematics has received considerable attention as new phylogenetic and geographical evidence has become available. Aside from the descriptions of new genera, species, and subspecies (Table 1), the most noteworthy action was the split of the genus Python by Rawlings et al. (2008), placing two Asian taxa, *reticulatus* and *timoriensis*, into Broghammerus.

**Table:** **Table 1.** New and resurrected taxa from 1999 until 2010. Numbers in parentheses represent the number of taxa deemed unavailable.

*Year*	*New genera*	*Resurrected genera*	*New species*	*Resurrected species*	*Elevated to species rank*	*New subspecies*	*Resurrected subspecies*
1999	0	0	0	0	2	0	0
2000	2(1)	2	5	0	8	7	2
2001	0	0	0	0	3	0	0
2002	0	0	1	0	0	2	0
2003	0	(1)	1(2)	(1)	0	(3)	0
2004	2	4	2	1	0	9	0
2005	0	0	0	0	0	0	0
2006	0	0	0	0	0	0	0
2007	0	0	0	0	0	0	0
2008	0	0	3	0	3	0	0
2009	(2)	0	0	(2)	(1)1	(4)1	0
2010	0	0	0	0	0	0	0
Total*	**4**	**6**	**12**	**1**	**17**	**19**	**2**

* Total figures exclude those taxa considered unavailable.

One author, the amateur herpetologist Raymond T. Hoser of Victoria, Australia, has caused considerable confusion in python taxonomy over the last decade by describing numerous taxa (6 new genera and subgenera, 4 new species, and 19 new subspecies) in the non-peer-reviewed literature without providing adequate descriptions for his proposed new taxa (for discussions see Aplin 1999, 2002, Wüster et al. 2001, Williams et al. 2006, 2008, Schleip 2008). Hoser rarely included important taxonomic information or data on scale counts, numbers of specimen examined, statistics, or the results of DNA analysis. Moreover, Hoser designated several types without ever having apparently examined them. Although not mandatory, the International Code of Zoological Nomenclature (ICZN 1999), hereafter termed the “Code”, recommends that only specimens personally examined by the author should be designated as types (Recommendation 73B). Furthermore, Hoser himself (1996, 1997) considered failing to examine type specimens “sloppy taxonomy”. Another recommendation (Recommendation 73C), which states which data should be provided with the holotype, is often not followed by this author. In general, inadequate descriptions inevitably lead to problems in clearly assigning specimens to established taxa, and are expensive and time-consuming for subsequent workers who have to re-examine the type material in order to make taxonomic decisions, instead of being able to rely on adequate original descriptions.

In general, the professional herpetological community has rarely accepted Hoser's taxa (Wüster et al. 2001, Aplin 2002, Williams et al. 2006, 2008, Schleip 2008, Zaher et al. 2009) unless one of his numerous names turns out to be valid and a senior synonym based on more exacting scientific work carried out by professional researchers, as was the case with Broghammerus.

## Approach and Scope

The primary objective of this taxonomic checklist is to provide an overview of the taxa in the family Pythonidae, and to establish their nomenclatural status under the provisions of the Code and their current taxonomical status based on published works and knowledge. It is, however, beyond the scope of this list to propose re-classifications or re-arrangements of genera that lack fully resolved phylogenetic relationships. Although this checklist can only be a snapshot in time, it is intended to continue the work of McDiarmid et al. (1999) for the family Pythonidae over the past decade and provide updates to the list compiled by Henderson and Powell (2007). For taxa described during the past decade type species (for generic names) or type specimens (for specific names) are provided along with their type localities. Recently designated neotypes are also provided. Where new distributional information is available, this is included with the relevant citation. However, in contrast to the work of McDiarmid et al. (1999) and Henderson and Powell (2007), this checklist also contains extinct taxa.

Taxa are hierarchically arranged by indentation, and are presented in alphabetical order at the level of genera, species, and subspecies, although, in the case of subspecies, the nominate subspecies precedes other subspecies, which then are listed in alphabetical order. Annotations are made directly below the relevant taxon, unless otherwise stated. Synonyms before the year 1999, and remarks on valid taxa, unless new data are available, can be found in McDiarmidet al. (1999). A key to the extent genera, species and subspecies recognized within the family of Pythonidae is provided in Appendix 2.

### Interpretation and application of the Code

The Code rules on issues regarding nomenclatural acts and works, and aims to “provide the maximum universality and continuity in the scientific names of animals compatible with the freedom of scientists to classify animals according to taxonomic judgments” (ICZN 1999). Due to its universality, the wording of the Code leaves considerable room for interpretation. For the assessment of the nomenclatural status of published names, and for the purpose of nomenclatural stability, the Code was here strictly applied to all names. In any case of ambiguous wording, the authors have consulted the glossary of the Code as suggested in the Code’s “Explanatory Note”, and as stated in article 89. In the checklist we use the abbreviation “APP” (application):

#### APP1.

“Characters”: To be available a name must “be accompanied by a description or definition that states in words characters that are purported to differentiate the taxon” (ICZN 1999: Art. 13.1.1). A description in the meaning of the Code is “a statement in words of taxonomic characters of a specimen or a taxon” (ICZN 1999: glossary entry for “description”), and a definition is “a statement in words that purports to give those characters which, in combination uniquely distinguish a taxon” (ICZN 1999: glossary entry for “definition”). The glossary defines the word taxon as a “taxonomic unit, whether named or not: i.e., a population, or group of populations of organisms which are usually inferred to be phylogenetically related and which have characters in common which differentiate (…) the unit (e.g., a geographic population, a genus, a family, an order) from other such units” (ICZN 1999: glossary entry for “taxon”). This latter statement clearly excludes distribution itself as a character to differentiate taxa and that complies with article 13.1.1, since it requires characters to differentiate a “geographic population” from other such units. Many taxonomists are likely to accept a geographic population, especially an insular population, only separated from other such populations by distribution, at subspecific rank. However, the Code does not distinguish between specific and subspecific rank in its requirements (Arts. 45.1, 45.2), and therefore subspecies must also be distinguishable by characters other than by their isolated locality or distribution.

#### APP2.

“Generalized statements”: Generalized statements such as “separated by distribution” or “separated by analysis of DNA” or relative statements such as “usually (but not always) has” do not constitute a character in the sense of article 13.1.1 (APP1). Analysis of DNA clearly describes a method although genomic differences are of diagnostic value, and distribution itself is not a character, as it is not intrinsic to any specimen within the taxon. Therefore, these are not attributes of an organism (see glossary for character). Moreover, strictly following the glossary definition of the word description, the Code would require that a taxon must be uniquely distinguished from other taxa and generalized statements do not imply uniqueness.

#### APP3.

“Priority”: Article 23.3.5 requires the replacement of an unavailable name with the oldest available synonym (senior synonym).

#### APP4.

“Incorrect subsequent spelling”: Article 33.3 states that “any subsequent spelling of a name different from the correct original spelling, other than a mandatory change or an emendation, is an “incorrect subsequent spelling”; it is not an available name and, like an incorrect original spelling (…), it does not enter into homonymy and cannot be used as a substitute name” (ICZN 1999). For species-group names article 11.9.3.2 states that they are “deemed to have been published in combination with the correct original spelling of the generic name, even if it was actually published in combination with an emendation or incorrect spelling of the generic name” (ICZN 1999). Therefore, incorrect subsequent spellings are corrected to the original spelling.

#### APP5.

“*Nomen dubium*”(pl. *nomina dubia*): According to the glossary of the Code a *nomen dubium* is “a name of unknown or doubtful application” (ICZN 1999). This glossary definition leaves a wide scope for applying the term. A *nomen dubium* may be a lost type specimen or a type that lacks important diagnostic features so that a name cannot be applied to a specimen with clarity. (Melville (1980, 1984) noted that considering a name as *nomen dubium* is a matter of taxonomic decision and not a nomenclatural one. Moreover, Mones (1989) revealed that this term was first used for a taxon which was accompanied by an insufficient description. He states that the term “(…) denotes ignorance, incapability to interpret the facts, insufficient diagnosis, or actual poorness of the type specimen” (Mones, 1989: 232). We agree with the above mentioned views and, hence, insufficient information on the holotype (Recommendations 72E, 73A, see Introduction) that obviously was randomly chosen from an online database of a natural history museum and was not examined by the author (Recommendation 73B) along with an insufficient diagnosis or definition of taxonomic characters (see Art. 13.1.1, Recommendation 13A) may make a name be considered a *nomen dubium.* However, the name remains available, and a subsequent revision or re-description of the taxon may establish its validity.

#### APP6.

“*Nomen nudum*” (pl. *nomina nuda*):For generic names to be available, the Code requires “the fixation of a type species in the original publication” (ICZN 1999: Art. 13.3). All names must be “explicitly indicated as intentionally new” (ICZN 1999: Art. 16.1). Generic names, as well as specific and subspecific names, to which no characters were provided that comply with article 13.1.1 (see APP1, APP2), are deemed a *nomen nudum*, and therefore considered unavailable.

#### APP7.

“*Species inquirenda*”(pl. *species inquirendae*): This is “a Latin term meaning a species of doubtful identity requiring further investigation” (ICZN 1999: glossary entry).

#### APP8.

“Unavailable name”: A name is regarded as unavailable under the provisions of the Code, if either the requirements for publication or the requirements for availability are not met. This seems to be the case for names published by Hoser in his self-published Australasian Journal of Herpetology. Although the journal’s website states that several hard copies were placed in libraries to comply with the Code, these authors were unable to locate hard copies from any major European or North American library, or obtain such from the publisher when first issued (also see Recommendations 8B–D). An order form for hard copies (http://www.smuggled.com/AJHHCO1.htm, accessed 17 May 2009) was added to the publisher’s website on 7 May 2009. The National Library of Australia (NLA), the only library that lists this journal in their catalog, also has no hard copy (enquiry # NLAref21927, 16 April 2009) and only the PDF of the second issue (Hoser 2009) of the journal as of 17 May 2009 (NLA copy request CDC-10117150, 9.V.2009 could not be processed). Articles 8.1.2 and 8.1.3 of the Code state that to be regarded as published works they “must be obtainable, when first issued (…)”, and “must have been produced in an edition containing simultaneously obtainable copies by a method that assures numerous identical and durable copies”. Neither requirements was fulfilled. Later (20 May 2009), a colleague requesting original printed hard copies directly from the publisher only receiving single-sided, black and white versions of the online papers, printed on a domestic laser printer and bound by a large staple on the upper left hand corner (V. Wallach, pers. comm.). On his website, the publisher states “both print (first print run) and online are identical including use of color”. Therefore, the hard copy received by our colleague was apparently “printed on demand”. Article 9.7 states that “copies obtained on demand of an unpublished work [Art. 8], even if previously deposited in a library or other archive” do not constitute published work. The publisher disseminates the articles via the internet as PDFs downloadable from the journal’s website, and appears to rely on the trust of subsequent workers, that paper copies do exist (e.g., Zaher et al. 2009). However, the dissemination of PDFs over the internet does not currently constitute “published works” (Art. 9.6). Since no hard copies of the relevant second issue (Hoser 2009) were obtainable when first issued, and requested hard copies were “printed on demand”, this work must be regarded as “not published” under the provisions of the Code (Arts. 8.1.2, 8.1.3, 8.6, 9.6, 9.7) and the names therein are deemed unavailable (also see Wallach et al. 2009). The names, however, are listed for the completeness of the list but are not part of the formal synonymy.

## Abbreviations for Depositories of type material

AMAustralian Museum, Sydney, Australia

AMNHAmerican Museum of Natural History, New York, NY, USA

BPBMBernice P. Bishop Museum, Honolulu, Hawaii, USA

CASCalifornia Academy of Sciences, San Francisco, California, USA

FMNHField Museum, Chicago, IL, USA

MCZMuseum of Comparative Zoology, Harvard University, Cambridge, MA, USA

NMVMuseum of Victoria, Melbourne, Australia

MNHNMuséum national d’Histoire naturelle, Paris, France

QMQueensland Museum, Brisbane, Queensland, Australia

RMNHNaturalis, Leiden, The Netherlands

SAMASouth Australian Museum, Adelaide, South Australia, Australia

UTAUniversity of Texas at Austin, Austin, Texas, USA

WAMWestern Australian Museum, Perth, Western Australia, Australia

ZFMKZoologisches Forschungsinstitut und Museum Koenig, Bonn, Germany

ZMUCZoologisk Museum, Copenhagen, Denmark

## Checklist of the Pythonidae

### Genus Antaresia

Wells & Wellington, 1984

#### Remarks:

Cogger (2000) did not recognize this genus but placed the four species recognized therein without subspecies in the genus Liasis Gray.

#### Antaresia childreni

(Gray, 1842)

#### Antaresia maculosa

(Peters, 1873)

##### Synonyms: 

Antaresia maculosa brentonoloughlini Hoser, 2004

##### Distribution:

O’Shea et al. (2004) reported the first occurrence outside Australia, at Weam, Western Province, Papua New Guinea.

##### Antaresia maculosa brentonoloughlini

Hoser, 2004 [synonym of Antaresia maculosa]

###### Holotype:

AM R16772.

###### Type locality:

16 km east of Coen, Queensland, Australia.

###### Remarks:

Hoser (2004) separated this taxon from the nominate subspecies “by its greater preponderance of light colouration relative to dark blotches on the dorsal surface” (Hoser 2004), stating that the nominate form would “have roughly half to half (50:50) dark versus light blothes” whereas the ratio in this taxon “is generally at least 60% light colour to 40% or less darker blotches” (Hoser 2004), and by larger average size.

#### Antaresia perthensis

(Stull, 1932)

#### Antaresia stimsoni

(LA Smith, 1985)

##### Antaresia stimsoni stimsoni

(LA Smith, 1985)

##### Antaresia stimsoni campbelli

Hoser, 2000 [synonym of Antaresia stimsoni orientalis]

###### Holotype:

AM R69087.

###### Type locality:

Wilcannia, New South Wales, Australia.

###### Remarks:

The holotype of this taxon is also the paratype of Antaresia stimsoni orientalis Smith, 1985. Hoser (2000) separated this taxon from “other subspecies” by distribution (APP1, APP2), and from “other Antaresia stimsoni” by color. Hoser (2000) cited without acknowledgment a statement made by Ehmann (1992) and quoted by Kend (1997: 148) and added to the statement, “the snout has a less box-like anterior when compared with other Antaresia stimsoni” (Hoser 2000). However, since he considered Antaresia stimsoni a synonym of “Antaresia saxacola” (see comments on Antaresia stimsoni orientalis) and Antaresia stimsoni stimsoni a separate subspecies, it is not clear what he means by Antaresia stimsoni. This taxon is placed in the synonymy of Antaresia stimsoni orientalis until further research has assessed its validity. Subsequent workers (e.g., Sonnemann 2007) have not recognized this taxon.

##### Antaresia stimsoni orientalis

(LA Smith, 1985)

###### Synonyms:

Antaresia stimsoni campbelli Hoser 2000

###### Remarks:

Hoser (2000) resurrected the name Antaresia saxacola Wells & Wellington, 1985, but, contrary to Hoser’s (2000) claims that Antaresia stimsoni Smith, 1985 (Hoser provided an incorrect date: Smith 1995) would be a subjective junior synonym of Antaresia saxacola orientalis Smith, 1985 (see Shea and Sadlier, 1999), the date of publication for Smith (1985) preceded Wells and Wellington (1985) as stated by Wells (2009), which makes Antaresia saxacola a subjective junior synonym of Antaresia stimsoni. Nevertheless, because Wells and Wellington did not provide a description for Antaresia saxacola, the name was considered a *nomen nudum* by Underwood and Stimson (1990) and Shea and Sadlier (1999). We agree with these authors in considering “*saxacola*” a *nomen nudum* (APP6), and *orientalis* replaces it (APP3). Antaresia stimsoni orientalis was not listed by Cogger (1992), Barker and Barker (1994), Cogger (2000) and Henderson and Powell (2007), but was recognized by others (e.g., Ehmann 1992, Kend 1997, Walls 1998, Torr 2000, Wilson and Swan 2008).

### Genus Apodora

Kluge, 1993

#### Remarks:

Kluge (1993) established this monotypic genus for the species *papuana*, which until then was included in the genus Liasis Gray as Liasis papuanus. Because of the lack of clarity concerning the phylogenetic position of this taxon (Liasis, Apodora (Morelia, Python)) (see Kluge 1993: fig. 28), Kluge (1993: 53) characterized it with the term “*sedis mutabilis*” (of changing phylogenetic position, *sensu* Wiley, 1981, convention 4). However, recent researchers found some of the anatomical and morphological characters used in previous phylogenetic studies unsuitable due to misidentification (e.g., Scanlon 2001) or homoplasy (e.g., Wilcox et al. 2002, for detailed discussion see Rawlings et al. 2008). Scanlon (2001) analyzed a modified version of the dataset used by Kluge (1993) and found the genus Liasis to be paraphyletic (see Scanlon 2001: fig. 26). Later Rawlings et al. (2004) placed Apodora papuana as the sister clade to all other species of Liasis, which supports the separation of Apodora papuana as proposed by Kluge (1993), but this position was not well supported by their data. More recently, Rawlings et al. (2008) indicated a relationship between Apodora papuana and Liasis olivaceus within the monophyletic genus Liasis, both taxa forming a sister clade to the Liasis fuscus/*mackloti* complex, but again this position was not well supported. Hence, in anticipation of more robust data, we retain the current placement of this genus.

#### Apodora papuana

(Peters & Doria, 1878)

##### Synonyms:

Liasis papuanus Peters & Doria – Hoser 2000, 2004

Apodora papuana (Peters & Doria) – Rawlings and Donnellan 2003; Henderson and Powell 2007; Rawlings et al. 2008

### Genus Aspidites

Peters, 1877

#### Remarks:

Henderson and Powell (2007) and Swan (2007) did not list subspecies within Aspidites. Aspidites was considered most primitive within the Pythonidae (e.g., Stimson and Underwood 1990, Kluge 1993) due to a lack of thermoreceptive pits in the labial scales. However, current research (Westhoff and Collin 2008) has revealed that Aspidites possesses a single thermoreceptive pit within the rostralia.

#### Aspidites melanocephalus

(Krefft, 1864)

##### Synonyms:

Aspidites melanocephalus adelynensis Hoser, 2000

Aspidites melanocephalus davieii Hoser, 2000

Aspidites melanocephalus rickjonesii Hoser, 2009 (unavailable name, APP8)

##### Aspidites melanocephalus adelynensis

Hoser, 2000 [synonym of Aspidites melanocephalus]

###### Holotype:

WAM R51208 (see remarks).

###### Type locality:

Wyndham, Western Australia.

###### Remarks:

Hoser (2000) provided the same erroneous accession number for the holotype as was already provided by Smith (1985) in his original description; WAM R51208 is the number for a skink, Eremiascincus isolepis (*fide* Mecke et al. 2009) (Doughty, pers. comm.). Hoser (2000) separated this taxon from the nominate form by lower loreal, subocular, and parietal scale counts (see Barker and Barker 1994: 1–2). The same is stated to be diagnostic for Aspidites melanocephalus davieii, which makes them indistinguishable from each other, as already noted by Aplin (2002: 55–56) who considered Aspidites melanocephalus adelynensis the senior synonym due to page priority. The name is placed in the synonymy of Aspidites melanocephalus until further research can clarify its taxonomic position.

##### Aspidites melanocephalus davieii

Hoser, 2000 [junior synonym of Aspidites melanocephalus adelynensis and of Aspidites melanocephalus]

###### Holotype:

WAM R46170.

###### Type locality:

Tom Price, Western Australia.

###### Remarks:

Contrary to Aplin (2002: 56), we do not consider this name a *nomen nudum* because Hoser (2000) provided characters that purport to differentiate it from the nominate form. However, based on Hoser’s (2000) description, this taxon is indistinguishable from Aspidites melanocephalus adelynensis (see comments above). The name is therefore considered a subjective junior synonym of Aspidites melanocephalus adelynensis and is placed into the synonymy of Aspidites melanocephalus.

##### Aspidites melanocephalus rickjonesii

Hoser, 2009 [unavailable name (APP8)]

###### Holotype:

WAM 46170.

###### Type locality:

Tom Price, Western Australia.

###### Remarks:

Hoser (2009) had designated the same holotype and paratypes as for Aspidites melanocephalus davieii. The name is considered “not published” under the provisions of the Code (APP8) but would nevertheless be an objective junior synonym of Aspidites melanocephalus davieii.

#### Aspidites ramsayi

(Macleay, 1882)

##### Synonyms:

Aspidites ramsayi panoptes Hoser, 2000

Aspidites ramsayi richardjonesii Hoser, 2000

Aspidites ramsayi neildavieii Hoser, 2009 (unavailable name, APP8)

Aspidites ramsayi neildavieii Hoser, 2009 [unavailable name (APP8)]

##### Holotype:

WAM 34070.

##### Type species:

near Port Hedland, Western Australia.

##### Remarks:

Designation of the same type as for Aspidites ramsayi richardjonesi. The name is considered “not published” under the provisions of the Code (APP8) but would nevertheless be an objective junior synonym of Aspidites ramsayi richardjonesi.

##### Aspidites ramsayi panoptes

Hoser, 2000 [synonym of Aspidites ramsayi]

###### Synonyms:

Aspidites ramsayi richardjonesii – Hoser 2000

###### Holotype:

WAM R43459.

###### Type locality:

Burracoppin, Western Australia.

###### Remarks:

Distinguished from “the main race” by lower average ventral and subcaudal scale counts (citing Barker and Barker [1994: 5] in support of this claim), color darkening above the eye in adults, and “from all other Womas by distribution” (Hoser 2000: 10) (APP1, APP2). Because of the vague description of this taxon, specimens cannot be unambiguously assigned to this taxon. The name is placed into the synonymy of Aspidites ramsayi. For further comments see Aspidites ramsayi richardjonesii.

##### Aspidites ramsayi richardjonesii

Hoser, 2000 [junior synonym of Aspidites ramsayi panoptes]

###### Holotype:

WAM R34070.

###### Type locality:

near Port Hedland, Western Australia.

###### Remarks:

Aplin (2002) considered this taxon a *nomen nudum*. We disagree because Hoser (2000) provided characters that purport to differentiate this taxon from the “main race”. Nevertheless, this taxon is indistinguishable from Aspidites ramsayi panoptes, as both taxa share the diagnostic characters and are only separated by “vast distance” (Hoser 2000) (APP1, APP2) (also see Wüster et al. 2001). Without further data, these taxa must be treated as synonyms, with Aspidites ramsayi panoptes having priority.

### Genus Aspidoboa

Sauvage, 1884 [synonym of Python]

#### Remarks:

Hoser (2004) resurrected this genus to include the species of the Python curtus complex (*sensu* Keogh et al. 2001). As demonstrated by Rawlings et al. (2008) after exclusion of the taxa *reticulatus* and *timoriensis* (see Broghammerus), the genus Python forms a monophyletic grouping, including the taxon *brongersmai.* Since Keogh et al. (2001) demonstrated that *brongersmai* is the sister taxon to *curtus* and *breitensteini,* separating these three taxa from the genus Python would result in the non-monophyly of the genus. It is our opinion that the recognition of Aspidoboa at subgeneric rank only causes confusion and is unnecessary in a low-diversity genus as Python.

### Genus Australiasis

Wells & Wellington, 1984 [synonym of Morelia]

#### Synonyms:

Austroliasis Hoser, 2000 (incorrect subsequent spelling, APP4)

#### Remarks:

Hoser (2004) used the correct spelling rather than his earlier incorrect spelling of this taxon as “Austroliasis” (see below) but also included the species of the *amethistina*-complex (*sensu* Harvey et al. 2000) and furthermore added *timorensis* (APP4) Peters, 1877. Nevertheless, Hoser only listed this genus without comment or evidence for its resurrection.

#### Australiasis amethystinus

(Schneider, 1801) [synonym of Montypythonoides amethistina]

##### Australiasis amethystinus clarki

(Barbour, 1914) [synonym of Morelia amethistina]

###### Synonyms:

Austroliasis amethystinus clarki (Barbour) – Hoser 2000 (APP4)

Australiasis amethystina clarki (Barbour) – Hoser 2004 (APP4)

Australiasis clarki (Barbour) – Hoser 2009 (APP8, see introduction)

###### Remarks:

Hoser (2000) resurrected this taxon from the synonymy of Morelia amethistina and placed it along with the nominal form *amethistina amethistina* and the taxon *timorensis* Peters, 1877 (APP4, incorrect subsequent spelling of *timoriensis* Peters, 1877) into the genus “Austroliasis”. This generic name constitutes an incorrect subsequent spelling (APP4) of the genus Australiasis Wells and Wellington 1984. Harvey et al. (2000) examined the holotype of Liasis clarki Barbour and found it to be “conspecific with Morelia amethistina, rather than Morelia kinghorni” (Harvey et al. 2000: 155) and documented that “at least some snakes from [the Torres Strait] islands are Morelia amethistina (e.g., the Murray Islands where the type Liasis clarki was collected)” (Harvey et al. 2000: 162). Until further studies have evaluated the taxonomic status of this population, this taxon is placed in the synonymy of Morelia amethistina. Henderson and Powell (2007) did not recognize this taxon.

##### Australiasis amethystinus duceboracensis

(Günther, 1879) [synonym of Morelia amethistina]

###### Remarks:

Hoser (2004) listed this taxon for the population referred to as Morelia amethistina from New Ireland, Bismarck Archipelago, Papua New Guinea (see remarks on Morelia amethistina and Morelia clarki) without justification. Until further research has been carried out into the status of pythons in the Bismarck Archipelago, this species is herein assigned to the synonymy of Morelia amethistina.

### Genus Austroliasis

Hoser, 2000 [incorrect subsequent spelling of Australiasis Wells & Wellington, 1984 (APP4)]

#### Remarks:

Hoser (2000) intending to resurrect Australiasis Wells & Wellington, 1984 created an incorrect subsequent spelling. Under the rules of the ICZN, this name is not an available name (Art. 33.3, ICZN 1999). See Australiasis.

### Genus Bothrochilus

Fitzinger, 1843

#### Remarks:

Rawlings et al. (2008) identified a sister-group relationship of this monotypic genus with Leiopython, which they also considered monotypic. They proposed synonymy of Leiopython with Bothrochilus, with the latter being the senior synonym. Also see comments on Leiopython.

#### Bothrochilus boa

Fitzinger, 1843

### Genus Broghammerus

Hoser, 2004 fideRawlings et al. (2008)

#### Type species:

Python reticulatusSchneider, 1801

#### Remarks:

Subsequent workers did not recognize Broghammerus until a new analysis combining morphological and molecular evidence (Rawlings et al. 2008) led to a split of the genus Python. The latter authors expanded Hoser’s original concept of the genus to include the taxon *timoriensis* Peters, since they demonstrated that this species is more closely related to Broghammerus reticulatus than species retained within the genus Python (or to Austroliasis [i.e., Morelia], the genus to which Hoser assigned *timoriensis*).

#### Broghammerus reticulatus

(Schneider, 1801)

##### Neotype:

ZFMK 32378. Type locality: Rengit, West Malaysia; designated by Auliya et al. (2002).

##### Broghammerus reticulatus reticulatus

(Schneider, 1801)

###### Synonyms:

Python reticulatus reticulatus (Schneider) – Auliya et al. 2002

Broghammerus reticulatus dalegibbonsi Hoser, 2004

Broghammerus reticulatus euanedwardsi Hoser, 2004

Broghammerus reticulatus neilsonnemani Hoser, 2004

Broghammerus reticulatus patrickcouperi Hoser, 2004

Broghammerus reticulatus stuartbigmorei Hoser, 2004

###### Distribution:

For records in western Thailand, see Pauwels et al. (2003). O’Shea and Lazell (2008) reported a specimen from Itbayat Island, Batanes Province, Philippines, the northeastern-most record for the taxon.

###### Remarks:

Hoser (2004) describes the nominate form as “largish regional race with brownish head, much the same colour as the lighter dorsal body markings, although light-headed specimens are known and several colour variants and distinct colour mutations are also known”.

##### Broghammerus reticulatus dalegibbonsi

Hoser, 2004 [synonym of Broghammerus reticulatus reticulatus]

###### Holotype:

FMNH 142320.

###### Type locality:

Ambon, Makulu (=Moluccas), Indonesia.

###### Remarks:

Hoser (2004) asserts that size and color separate this subspecies from the nominate subspecies. The statement “generally smaller race” is as unspecific as the statement made for the size of the nominate form (see remarks there). Therefore, a differentiation of both based on size is nearly impossible. Hoser (2004) describes the color of this subspecies by stating “it rarely has a head lighter than the body as in some other variants of Broghammerus, such as those from Bali or parts of Thailand” (Hoser 2004). Based on this statement, this subspecies is indistinguishable from the nominate form (see remarks for Broghammerus reticulatus). No other characters are provided. Simply stating “best separated from all other Broghammerus by DNA analysis and/or accurate distribution information” does not constitute a statement of characters (APP1, APP2). The name is placed into the synonymy of the nominate form.

##### Broghammerus reticulatus euanedwardsi

Hoser, 2004 [nomen dubium, synonym of Broghammerus reticulatus reticulatus]

###### Holotype:

FMNH 180232.

###### Type locality:

Nakhom Ratchasima, Central Thailand.

###### Remarks:

Hoser (2004) separated this subspecies from the nominate subspecies by size, stating that this would be “a large race”. However, the nominate form was also claimed to be “largish”, hence, the former statement cannot separate this taxon from the nominate form. No further characters are provided to separate this taxon from other subspecies. The name is herein treated as *nomen dubium* (APP5) and is assigned to the synonymy of the nominate form.

##### Broghammerus reticulatus haydnmacphiei

Hoser, 2004 [synonym of Broghammerus reticulatus reticulatus]

###### Holotype:

FMNH 148968.

###### Type locality:

Sarawak, Borneo, West Malaysia.

###### Remarks:

In the original description, Hoser (2004) violated the Principle of Binominal Nomenclature (Arts. 5.2, 11.4.2) (Broghammerus reticulatus haydn macphiei). In accordance with articles 11.9.5 and 32.5.2.2, the name was corrected to comply with this principle.The author separatesthis taxon from the nominate form (referred to as “normal *reticulatus*”) only by “larger average adult size” (Hoser 2004) (alsosee comments for Broghammerus reticulatus euanedwardsi). Therefore, this taxon is indistinguishable from Broghammerus reticulatus euanedwardsi or from Broghammerus reticulatus reticulatus and is placed in the synonymy of the latter. Auliya et al. (2002) demonstrated that specimens from Bali, West Malaysia, Java, West Kalimantan, and Vietnam form a clade.

##### Broghammerus reticulatus jampeanus

(Auliya et al., 2002)

###### Holotype:

ZFMK 73475.

###### Type locality:

Tanahjampea Island, Indonesia.

###### Remarks:

This subspecies was recognized by De Lang and Vogel (2006), and O’Shea (2007), but was overlooked by Henderson and Powell (2007) (Henderson 2009, pers. comm.). However, the relevant paper was cited in the list of references by the latter authors.

##### Broghammerus reticulatus neilsonnemani

Hoser, 2004 [synonym of Broghammerus reticulatus reticulatus]

###### Holotype:

FMNH 53272.

###### Type locality:

Davao Province, Mindanao Island, Philippine Islands.

###### Remarks:

In the diagnosis for this taxon, Hoser (2004) claimed that this taxon attains larger size and stated the same as for Broghammerus reticulatus dalegibbonsi regarding the color (see remarks there). Since both are also true for the nominate form, and no further diagnostic characters are given, this taxon is placed in the synonymy of Broghammerus reticulatus reticulatus.

##### Broghammerus reticulatus patrickcouperi

Hoser, 2004 [synonym of Broghammerus reticulatus reticulatus]

###### Holotype:

MCZ R-25266.

###### Type locality:

“Djamplong”, South Timor, Indonesia. The MCZ online collection database provides the following information on the locality: “Djamplong, S Timor Indoaustralia, Indonesia, Timor Timur?, Nusa Tenggara”.

###### Remarks:

Hoser (2004) separated this subspecies from the nominate subspecies, referred to as “typical *reticulatus*”, by color, stating that this taxon is “usually a brightly coloured subspecies” (Hoser 2004). However, the author clearly stated that several color variants are know within the nominate form as well. Hoser did not provide other characters that would indicate whether the specimen is assignable to this taxon or the nominate form. The name is assigned to the synonymy of Broghammerus reticulatus reticulatus.

##### Broghammerus reticulatus saputrai

(Auliya et al., 2002)

###### Holotype:

ZFMK 73473.

###### Type locality:

Selayar Island, Indonesia.

###### Remarks:

Although this taxon was recognized by subsequent workers (e.g., De Lang and Vogel 2006, O’Shea 2007), it was overlooked by Henderson and Powell (2007) (Henderson 2009, pers. comm.). However, the latter authors cited the relevant work in the list of references.

##### Broghammerus reticulatus stuartbigmorei

Hoser, 2004 [nomen dubium, synonym of Broghammerus reticulatus reticulatus]

###### Holotype:

MCZ R-8003.

###### Type locality:

Buitenzore (believed a misspelling of Buitenzorg, the Dutch colonial name for Bogor), Java, Indonesia.

###### Remarks:

Hoser (2004) provided characters to separate this taxon, but he attempts to distinguish this subspecies from the species “Broghammerus reticulatus”, which includes the subspecies itself. This taxon is indistinguishable from other subspecies based on the original description and is therefore treated as *nomen dubium* (APP5), and placed in the synonymy of the nominate form.However, specimens from Bali, West Malaysia, Jaya, West Kalimantan, and Vietnam form a clade as demonstrated by Auliya et al. (2002).

#### Broghammerus timoriensis

(Peters, 1876)

##### Synonyms:

Austroliasis timorensis (Peters) – Hoser 2000 (incorrect subsequent spelling of Australiasis Wells & Wellington and of *timoriensis* Peters, APP4)

Australiasis timorensis (Peters) – Hoser 2004 (incorrect subsequent spelling of *timoriensis*, APP4)

Python timorensis (Peters) – Henderson and Powell 2007 (incorrect subsequent spelling of *timoriensis*, APP4)

Broghammerus timoriensis (Peters)– Rawlings et al. 2008

##### Remarks:

Doubts were casted in literature that this species occurs on Timor (e.g., Barker and Barker 1996, McDiarmid et al. 1999, O’Shea 2007) because no records other than the type specimen are known from Timor, and this reported occurrence is likely incorrect.

### Genus Chondropython

Meyer, 1874 [synonym of Morelia]

#### Remarks:

Hoser (2000) resurrected this genus for *viridis*, and later added two new subspecies *viridis shireenae* Hoser 2004 and *viridis adelynhoserae* Hoser 2009 (APP8). Rawlings et al. (2008) demonstrated that two lineages (“northern” and “southern” lineage) along with the taxon Morelia carinata, form asubclade within the clade that represented Morelia. Although Chondropython is the oldest available name for this subclade, we see no advantage in resurrecting taxa as subgenera in a low-diversity genus.

#### Chondropython azureus

(Meyer, 1874)

##### Synonyms:

Chondropython viridis (Schlegel, 1872) – Hoser 2000 (part)

Chondropython viridis viridis (Schlegel) – Hoser 2004 (part)

Morelia viridis (Schlegel) – Henderson and Powell 2007 (part)

Morelia azurea (Meyer, 1874) – this paper

##### Remarks:

Resurrected from the synonymy of Morelia viridis by Hoser (2009). Rawlings and Donnellan (2003) revealed the existence of a sibling species pair within the green tree python. The authors found a genetic divergence of about 7% in mitochondrial DNA (cytochrome b gene) between the northern and southern lineages, separated by the Central Mountain Range that extends in an east-west direction through New Guinea (also see comments on Morelia viridis). Rawlings and Donnellan (2003) revealed the existence of two species, one from north of the central cordillera, the other from the south, including the Aru Island and Australian populations. Nevertheless, within the southern lineage the Australian material formed a well supported clade whereas material from Aru Island clustered with that from Merauke and Timika. The authors state that “a determination of the species status of the northern and southern lineages awaits a more thorough assessment of divergence at nuclear genes based on wider geographic sampling than we could achieve herein with allozymes” (Rawlings and Donnellan 2003: 42). In 2008, Rawlings et al. (2008: 604) referred to the northern populations as the “unnamed sibling taxon of Morelia viridis”. However, it is not yet evident that only a single taxon occurs on Aru Island, and that the published type locality for Morelia viridis is correct. The name *azureus* Meyer 1874 would be available for the northern linage, having its type locality on Biak Island, one of the localities from which specimens of “Morelia viridis N[orth]” were analyzed by Rawlings et al. (2008) and hence a strong candidate for the taxon name, based on priority. Since the types are presumed lost, we call for the designation of a neotype.

#### Chondropython viridis

Schlegel, 1872 [synonym of Morelia viridis]

##### Chondropython viridis adelynhoserae

Hoser, 2009 [unavailable name (APP8, see introduction)]

###### Holotype:

AM R129716.

###### Type locality:

Normanby Island, d’Entrecasteaux Archipelago, Milne Bay Province, Papua New Guinea.

###### Remarks:

Rawlings and Donnellan (2003) revealed a genetic distance of about 3% in mitochondrial DNA (cytochrome b gene) between the Normanby Island specimen and all other specimens examined from the southern parts of New Guinea. However, this analysis was based on a single museum specimen from Normanby Island. Further research is needed to ascertain the taxonomic status of this population. However, this name is considered unavailable (APP8, also see introduction).

##### Chondropython viridis shireenae

Hoser, 2004 [synonym of Morelia viridis]

###### Holotype:

NMV D51862.

###### Type locality:

Cape York, Queensland, Australia.

###### Remarks:

Hoser (2004) stated that the “white or other markings along the vertebra” are not diagnostic for this subspecies, but that “a very thin line or line of dots along the spine” is “generally a diagnostic trait for adults of this subspecies”, although the author further states, that he had seen specimens with and without such markings. Furthermore, he noticed that “vertebral markings decline with age”. With the latter comments, the author himself invalidated the utility of vertebral markings as a diagnostic character. In the absence of other characters, this taxon is apparently indistinguishable from the nominate form. Furthermore, as demonstrated by Rawlings and Donnellan (2003: 36), “all of the Australian haplotypes, which form a single lineage, are nested among the southern New Guinean haplotypes“. We therefore placed this taxon in the synonymy of Morelia viridis (see comments there).

### Genus Heleionomus

Gray, 1842 [synonym of Python]

#### Synonyms:

Helionomous Gray, 1841 (*nomen nudum*) – Hoser 2004 (*nomen nudum* APP6)

#### Remarks:

The type species for Heleionomus Gray, 1842 is Heleionomus variegatus [= Python natalensis]. The resurrection of the genus Heleionomus for Python sebae and Python natalensis is unwarranted because the actual status of *natalensis* and *sebae* has not been fully resolved and, furthermore, separation from Python would compromise monophyly of the genus Python. Rawlings et al. (2008) showed a sister-group relationship between *sebae* and *molurus* and that the genus Python (after exclusion of *reticulatus* and *timoriensis*) forms a monophyletic group. This genus is therefore assigned to the synonymy of Python.

### Genus Helionomus

Gray, 1842 [nomen nudum (APP6), incorrect subsequent spelling (APP4)]

#### Remarks:

Hoser (2004) obviously intended to resurrect the genus Heleionomus Gray, 1842 but changed the name to “Helionomus”. This constitutes an incorrect subsequent spelling (Art. 33.3). However, the name Helionomus was already used by Gray (1841) listed in the index for Boidae, but no species was assigned to this name, and it is therefore considered a *nomen nudum*. Also see Heleionomus.

### Genus Jackypython

Hoser, 2009 [unavailable name (APP8)]

#### Type species:

Python carinatus Smith, 1980

#### Remarks:

Hoser (2009) introduced this name as a subgenus of Morelia Gray to include the single species Morelia carinata.

### Genus Katrinus

Hoser, 2000 [junior synonym of Liasis]

#### Type species:

Liasis fuscus Peters, 1873

#### Remarks:

Hoser (2000) established this genus for the separation of the water pythons (Liasis fuscus and Liasis mackloti) from the olive pythons (Liasis olivaceus), both currently referred to Liasis. He distinguished the two genera by the number of mid-body rows, stating that “Liasis usually has over 60” (Hoser 2000) (APP2). Barker and Barker (1994: 35) provided a range of 58–63 mid-body scale rows for Liasis olivaceus barroni. According to Rawlings et al. (2004, 2008), the taxa *fuscus* and *mackloti* are closely related to each other and since Liasis mackloti Duméril & Bibron is the name-bearing type of Liasis (by subsequent designation [see Stimson and McDowell (1986) and Opinion 1514, ICZN 1988]) and because Gray (1849) had proposed the subgeneric name Lisalia for Liasis olivaceus as well as Simalia (in part) for Liasis mackloti, the genus Katrinus must be considered a subjective junior synonym of Simalia, which itself is a synonym of Liasis Gray. See comments on the genus Apodora. Subsequent workers have not recognized Katrinus as a valid taxon.

#### Katrinus fuscus

(Peters, 1873) Hoser, 2000 [junor synonym of Liasis fuscus]

##### Katrinus fuscus cornwallisius

(Günther, 1879) [junior synonym of Liasis fuscus]

###### Type locality:

Dauan (as Cornwallis) Island, Torres Straits, Australia.

###### Remarks:

Katrinus cornwallisius Günther, 1879 was resurrected from the synonymy of Liasis fuscus by Hoser (2000) for the Torres Strait islands and New Guinean populations. However, Rawlings et al. (2004) demonstrated that specimens from Queensland, the Torres Strait islands (Saibai), and New Guinea form a well-supported clade, which was considered the sister group to the clade comprising the Northern Territory and Indonesian populations. Since Queensland is the type locality of Liasis fuscus Peters, the resurrection of this name is unwarranted as it is a junior synonym to Liasis fuscus. The name *cornwallisius* is therefore placed into the synonymy of Liasis fuscus.

##### Katrinus fuscus jackyae

(Hoser, 2004) [nomen dubium, synonym of Liasis fuscus]

###### Holotype:

WAM R13882.

###### Type locality:

Kalumburu, Western Australia.

###### Remarks:

Hoser (2004) claimed that several diagnostic features separated this taxon from others, but discusses only one (APP2); he stated that “in Katrinus fuscus fuscus (from coastal Queensland) the upper lips are pale with a little brown peppering. However, in Katrinus fuscus jackyae (from the NT and WA) the lips are usually darker with more dark brown peppering or even blotches” (Hoser 2004). He continued that this subspecies would intergrade with Katrinus fuscus cornwallisius around the Gulf of Carpentaria. The name is herein considered a *nomen dubium* (APP5).

### Genus Leiopython

Hubrecht, 1879

#### Remarks:

Recent studies revealed that Bothrochilus and Leiopython form a clade. Thus, since Rawlings et al. (2008) considered both genera monotypic, they proposed “the use of a single generic name (Bothrochilus) for this species pair” (Rawlings et al. 2008: 613). Later, Schleip (2008) demonstrated that this genus is not monotypic. Rawlings et al. (2008) had used material from Leiopython hoserae for their genetic analysis (GeneBank accession number U69835, Western Province, PNG at Mawatta). Until further molecular genetic data clarify the relationships of the taxa involved, and in deference to nomenclatural stability, we are reluctant to synonymize Leiopython with Bothrochilus.

#### Leiopython albertisii

(Peters & Doria, 1878)

##### Synonym:

Leiopython albertisii barkeri Hoser, 2000 (*nomen nudum*, APP6, see below)

Bothrochilus albertisii (Peters & Doria) – Rawlings et al. 2008

Leiopython albertisi barkerorum Hoser – Hoser 2009 (unavailable name, APP8)

##### Distribution:

Mulyadi (2007) reported the occurance of Leiopython albertisii from Lopintol (Waigeo) and Schleip (2008) from Emirau Island, St. Matthias Group, Bismarck Archipelago, New Ireland Province, Papua New Guinea.

##### Remarks:

Henderson and Powell (2007) listed only Leiopython albertisii Peters & Doria, 1878. Hoser (2000) incorrectly ascribed *albertisii* to Gray 1842. The taxon was named in honor of Italian naturalist Luigi Maria D’Albertis, who made a name for himself in New Guinea. D’Albertis was only a few months old in 1842 and would, therefore, have been an unlikely recipient of Gray’s dedication. Furthermore, Hoser repeatedly used an incorrect spelling for the species *albertisii* by omitting the terminal –*i* (APP4, article 33.4).

##### Leiopython albertisii barkeri

Hoser, 2000 [nomen nudum (APP6)]

###### Synonyms:

Leiopython albertisi barkerorum Hoser, 2009 (APP4, APP8, see introduction)

###### Remarks:

Hoser (2000) differentiated this subspecies only by remote distribution (APP2). Other characters mentioned by Hoser (2000) were said to overlap with the nominate form. Wüster et al. (2001) and Schleip (2008) therefore considered the name a *nomen nudum*. Furthermore, since the name honours two persons, it should have been suffixed with –*orum.* In 2009 Hoser re-described this taxon with the name emended to *albertisi barkerorum.* However, the name is considered not published under the Code (APP8).

#### Leiopython bennettorum

Hoser, 2000 (name emended by Wüster et al. 2001)

##### Synonyms:

Leiopython albertisii bennetti Hoser 2000

Leiopython bennettorum Hoser – Schleip 2008 (name emended)

##### Holotype:

BPBM 5452.

##### Type locality:

near Wau, Morobe Province, Papua New Guinea.

##### Remarks:

The original spelling *bennetti* (Hoser 2000) was emended (Wüster et al. 2001, Schleip 2008) because the taxon honours two persons (Art. 31.1.2, ICZN 1999) and should have been suffixed with -*orum*, a correction proposed by Wüster et al. (2001) and subsequently corrected by Schleip (2008).

#### Leiopython biakensis

Schleip, 2008

##### Holotype:

RMNH 10193.

##### Type locality:

Biak Island.

#### Leiopython fredparkeri

Schleip, 2008

##### Holotype:

CAS 118906.

##### Type locality:

Karimui, Simbu Province, Papua New Guinea.

#### Leiopython hoserae

Hoser, 2000

##### Synonyms:

Leiopython albertisii (Peters & Doria) – Henderson and Powell 2007

Leiopython hoserae Hoser – Schleip 2008

##### Holotype:

AMNH R-107150.

##### Type locality:

Wipim, Western Province, Papua New Guinea.

#### Leiopython huonensis

Schleip, 2008

##### Holotype:

AMNH R-95535.

##### Type locality:

Lae, Huon Peninsula, Morobe Province, Papua New Guinea.

### Genus Lenhoserus

Hoser, 2000 [synonym of Morelia]

#### Type species:

Python boeleni Brongersma, 1953.

#### Remarks:

Hoser (2000) established thismonotypic genus for Morelia boeleni stating “while the Boelen’s Python (*boeleni*) has close affinities with the carpet pythons, there is no evidence before this author to suggest that the relationship is any closer than that between the Green (*viridis*) and carpets. Thus if *viridis* is entitled to be placed in a separate genus to the carpets, so too should be *boeleni*” (Hoser 2000: 21–22). Rawlings et al. (2008) supported monophyly of the genus Morelia including Morelia boeleni. Lenhoserus would therefore only be a subgenus within Morelia. Other authors have not adopted this name, and, in adherence to nomenclatural stability, we regard Lenhoserus as a subjective junior synonym of Morelia (see comments there).

### Genus Liasis

Gray, 1842

#### Synonyms:

Katrinus Hoser, 2000

#### Remarks:

Scanlon and Mackness (2002) considered the gender of Liasis Gray feminine because Gray’s (1842) original use of the combination Liasis olivacea implied it to be feminine. However, Gray (1842) also used the masculine gender for Liasis amethystinus [=Morelia amethistina (Schneider)] within Liasis. Hence, Gray did not clearly indicate his intentions concerning the gender of Liasis. Despite this discordance in gender, the ICZN had used it plenary power (Art. 81.1, ICZN 1999) to fix a type species for Liasis (Opinion 1514, ICZN 1988), and additionally (but perhaps not deliberately) fixed the gender as masculine. The name and gender also entered the List of Available Names in Zoology (also see Art. 80.6, 80.7, ICZN 1999). These authors therefore follow the predominant use of a masculine gender for Liasis.

#### Liasis dubudingala

Scanlon and Mackness, 2002 [extinct species]

##### Synonyms:

?Morelia sp. – Archer and Wade 1976

##### Holotype:

QMF 9132, mid-trunk vertebra.

##### Type locality:

Main Quarry, Allingham Formation (early Pliocene), Bluff Downs Station, northeastern Queensland.

##### Remarks:

Scanlon and Mackness (2002: 433) stated that “the limited number of characters identified here for pythonine vertebrae thus imply a position either within, or as a sister taxon to, Liasis (*sensu stricto*)”.

#### Liasis fuscus

Peters, 1873

##### Synonyms:

Katrinus fuscus (Peters) – Hoser 2000

Liasis fuscus Peters – Rawlings et al. 2004; Henderson and Powell 2007

Katrinus fuscus fuscus (Peters) – Hoser 2000

Katrinus fuscus cornwallisius(Günther, 1879) – Hoser 2000

##### Remarks:

Kluge (1993) synonymized Liasis fuscus Peters, 1873 with Liasis mackloti Duméril & Bibron, 1844. However, Rawlings et al. (2004) demonstrated that specimens from Queensland (the type locality of this taxon), the Torres Strait islands (Saibai), and New Guinea form a well-supported clade, which is considered the sister group to the clade comprising the Northern Territory and Indonesian populations. Also see comments on Katrinus. Henderson and Powell (2007) did not recognize any subspecies within Liasis fuscus, a position followed herein. Some authors refer to this taxon as Liasis mackloti Duméril & Bibron (e.g., Hay 2007).

#### Liasis mackloti

(Duméril & Bibron, 1844)

##### Synonyms:

Katrinus mackloti (Duméril & Bibron, 1844) – Hoser 2000

Liasis mackloti Duméril & Bibron – Rawlings et al. 2004; Henderson and Powell 2007

##### Remarks:

Hoser (2000) placed this species in his genus Katrinus (see comments there). The recognition of the subspecies Liasis mackloti dunni and Liasis mackloti savuensis is supported by Rawlings et al. (2004). Carmichael et al. (2007) provide additional evidence (different courtship behaviors and pheromone trailing) for this placement. See the latter citation for additional information on biogeography.

##### Liasis mackloti mackloti

(Duméril & Bibron, 1844)

###### Synonyms:

Katrinus mackloti mackloti (Duméril & Bibron) – Hoser 2000

Liasis mackloti mackloti Duméril & Bibron – Rawlings et al. 2004; Henderson and Powell 2007

##### Liasis mackloti dunni

Stull, 1932

###### Synonyms:

Katrinus mackloti dunni (Stull) – Hoser 2000

Liasis mackloti dunni Stull – Rawlings et al. 2004; Henderson and Powell 2007.

###### Remarks:

Carmichael et al. (2007) note that sexual dimorphism is found among Macklot’s pythons but it is different from Liasis mackloti mackloti and Liasis mackloti savuensis; in Liasis mackloti dunni males are larger than females and engage in male-male combat.

##### Liasis mackloti savuensis

(Brongersma, 1956)

###### Synonyms:

Katrinus savuensis (Brongersma) – Hoser 2000

Liasis mackloti savuensis Brongersma – Rawlings et al. 2004; Henderson and Powell 2007

###### Remarks:

Referred to as Liasis savuensis by some authors (Hoser 2000, Vidal et al. 2007).

#### Liasis olivaceus

Gray, 1842

##### Synonyms:

Liasis olivacea Gray, 1842 – Gray 1842

Morelia antiqua (Smith & Plane, 1985) – Kluge 1993

Liasis olivacea Gray, 1842 – Scanlon and Mackness 2002 (see remarks at Liasis)

##### Remarks:

We accept the subspecies proposed by Smith (1981) and supported by molecular genetic evidence from Rawlings et al. (2004).

##### Liasis olivaceus olivaceus

Gray, 1842

##### Liasis olivaceus barroni

LA Smith, 1981

### Genus Montypythonoides

Smith & Plane, 1985 [subjective junior synonym of Morelia]

#### Type species:

Montypythonoides riversleighensis Smith & Plane, 1985 [extinct species]

#### Remarks:

Smith and Plane (1985: 194) stated that this genus “…shows strong relationship with species of Morelia”. Also see Morelia riversleighensis.

### Genus Morelia

Gray, 1842

#### Synonyms:

Lenhoserus Hoser, 2000

Chondropython Meyer, 1874 – Hoser 2000

Nyctophilopython Wells & Wellington, 1985 – Hoser 2000

Montypythonoides Smith & Plane, 1985 – Scanlon 2001

Australiasis Wells & Wellington, 1984 – Hoser 2004

#### Remarks:

Hoser (2000) proposed the splitting of this genus into several genera. He created a new genus, Lenhoserus Hoser (see comments there) (for Morelia boeleni), and resurrected Australiasis Wells & Wellington (for Morelia amethistina and Broghammerus timoriensis), but created an unavailable name (APP6) (“Austroliasis”) by incorrect subsequent spelling (APP4). Later, Hoser (2004) used the correct spelling Australiasis Wells & Wellington, added the species recognized by Harvey et al. (2000) and additionally resurrected the taxon *duceboracensis* Günther 1879 (see comments there). Furthermore, he resurrected Chondropython Meyer 1874 (for Morelia viridis) and Nyctophilopython Wells & Wellington (for Morelia oenpelliensis). However, phylogenetic studies (Rawlings et al. 2008) revealed that this taxonomic action is unwarranted. Although Rawlings et al.’s (2008) maximum parsimony analysis showed Morelia to be diphyletic (but monophyletic in Bayesian analysis), the separation of the *amethistina*-complex (*sensu* Harvey et al. 2000) and of *oenpelliensis* from the *bredli*/*spilota*-clade would in any case be unwarranted and would nullify the monophyly of this grouping. The resurrection of Chondropython would only be warranted at subgeneric rank with the inclusion of the two recognized lineages of the green tree python (*sensu* Rawlings and Donnellan 2003) and of Morelia carinata. However, Rawlings and Donnellan (2003) and Rawlings et al. (2008) avoided such placement because the phylogeny was not fully resolved (see comments for Chondropython azureus) The placement of Morelia boeleni as a separate monotypic genus is also unwarranted. We do not see any value in dividing such a small genus, and in the interests of nomenclatural stability, we place Australiasis, Lenhoserus, Chondropython, and Nyctophilopython in the synonymy of Morelia.

#### Morelia amethistina

(Schneider, 1801)

##### Synonyms:

Austroliasis amethistina (Schneider) – Hoser 2000 (incorrect subsequent spelling, APP4)

Australiasis amethistina (Schneider) – Hoser 2004

Australiasis amethystina clarki (Barbour, 1914) – Hoser 2004

Australiasis duceboracensis  (Günther, 1879) – Hoser 2004

Australiasis amethistina (Scheider) – Hoser 2009 (APP8, see introduction)

Australiasis dipsadides (Ogilby, 1891) – Hoser 2009 (APP8, see introduction)

##### Distribution:

Kraus and Allison (2004) reported Morelia amethistina from Fergusson Island.

##### Remarks:

Harvey et al. (2000) identified three races within the species, two from the mainland of New Guinea, separated by the Central Mountain Range (also see remarks on Morelia clarki), and another race from New Ireland (see remarks on Morelia duceboracensis). This is consistent with other species found in this region (e.g., the two lineages of the green tree python (*sensu* Rawlings and Donnelan 2003), and Leiopython albertisii/Leiopython hoserae). According to Harvey et al. (2000), the holotype of Australiasis amethistina is lost. We call for the designation of a neotype.

#### Morelia antiqua

(Smith & Plane, 1985) [synonym of Morelia riversleighensis, extinct species]

##### Synonyms:

Morelia antiquus Smith & Plane, 1985 – Smith and Plane 1985

Morelia antiqua – Scanlon 1992 (*antiquus* amended for gender by Scanlon 1992)

Liasis olivacea Gray, 1842 – Kluge 1993

##### Holotype:

Commonwealth Paleontological Collection 25077 (not “20577”; see Scanlon 2001), right dentary.

##### Type locality:

Camfield Beds, Blast Site, Bullock Creek, Northern Territory, Australia. Late middle Miocene (Scanlon 2001).

#### Morelia riversleighensis

(Smith & Plane, 1985) – Scanlon, 2001

##### Remarks:

Smith and Plane (1985) documented significantly lesser curvature in the teeth of this taxon, to that found in species of the genera Python and Morelia, and because “…of the slight curvature of the dentary teeth…” (Smith and Plane 1985: 194) the authors considered this taxon more closely related to Morelia than to Python.

#### Morelia azurea

(Meyer, 1874)

##### Remarks:

See Comments on Chondropython azureus and Morelia viridis.

#### Morelia boeleni

(Brongersma, 1953)

##### Synonyms:

Lenhoserus boeleni (Brongersma) – Hoser 2000

Morelia boeleni (Brongersma) – Henderson and Powell 2007; Flagle and Stoops 2009

##### Remarks:

Austin et al. (2009) found little genetic divergence within specimens across the species’ range. A single specimen out of 98 examined using the cytochrome b gene, from the eastern Morobe Province, PNG showed about 1.1% genetic divergence from specimens from West Papua. This demonstrates reduced genetic diversity within this taxon.

#### Morelia bredli

(Gow, 1981)

##### Remarks:

Fyfe (2007) lists this species as subspecies Morelia spilota bredli.

#### Morelia carinata

(Smith, 1981)

#### Morelia clastolepis

Harvey et al., 2000

##### Synonyms:

Australiasis clastolepis (Harvey et al.) – Hoser 2004, 2009 (APP8, see introduction)

Morelia clastolepis Harvey et al. – Henderson and Powell 2007

##### Holotype:

UTA 44486.

##### Type locality:

Ambon, Maluku (= Moluccas), Indonesia.

#### Morelia kinghorni

Stull, 1933

##### Distribution:

For range extensions in Queensland see Augusteyn (2004) and Fearn and Trembath (2006).

#### Morelia macburniei

Hoser, 2004 [synonym of Morelia spilota imbricata]

##### Holotype:

SAMA R13994.

##### Type locality:

St. Francis Island, South Australia.

##### Remarks:

Hoser (2004) separated this taxon from its closest relative Morelia spilota imbricata (see Schwaner et al. 1988) on the ground of “higher incidence of scale anomalies” to the ventral scales. It can be argued that anomalies do not make good diagnostic characters, and these anomalies were already described in detail by Schwaner et al. (1988). Hoser (2004) further claims that this taxon may be distinguished from Morelia mippughae “by having lanceolate-shaped dorsal scales as opposed to more rhomboidal-shaped dorsal scales” (also see comments on Morelia mippughae). According to Schwaner et al. (1988: 15), and in support of Smith (1981), “specimens of *imbricata* have distinctly elongated, lanceolate-shaped, posterior dorsal scales. Morelia spilota variegata usually have the rhomboidal condition”. Furthermore, Schwaner et al. (1988) also reported that specimens from the St. Francis Island exhibited reduced ventral and subcaudal scale counts and a shorter tail than specimens from other populations. Hoser (2004) stated that this taxon is distinguishable from “all other Morelia by colouration and patterns” (Hoser 2004), but contradicted this statement when stating that this taxon is “highly variable in individual colouration and pattern”, and that “this species cannot be definitively separated from other Morelia on the basis of scalation alone as these properties (ventral counts and the like) may overlap with other Morelia” (Hoser 2004). Based on this description, it is unlikely that specimens can be correctly assigned to this species unless they were known to originate from the type locality. Schwaner et al. (1988: 14) and Pearson et al. (2002) assigned the St. Francis Island population to the subspecies Morelia spilota imbricata. We concur with this and relegate this taxon to the synonymy of Morelia spilota imbricata. Mense (2006), Henderson and Powell (2007), and Swan (2007) did not list this taxon.

#### Morelia mippughae

Hoser, 2004 [nomen dubium (APP5)]

##### Holotype:

SAMA R14261.

##### Type locality:

Iron Duchess, Middleback Ranges, South Australia.

##### Remarks:

Hoser (2004) separated this taxon from its relative Morelia macburniei “by a lower incidence of scale anomalies” (Hoser 2004) of the ventral scales. This is meaningless because most populations will show few anonomalies, hence, using the “normal state” as a character does not differentiate this taxon from others. Hoser (2004) continued that this taxon has “more rhomboidal-shaped dorsal scales as opposed to having lanceolate-shaped dorsal scales” (Hoser 2004), which is, according to Schwaner et al. (1988) also true for Morelia spilota variegata (also see remarks on Morelia macburniei). It is further separated from its closest relative Morelia spilota metcalfei by its color pattern and from all other Morelia by coloration and patterning. Hoser claimed that “a suite of characteristics” separate this taxon from its closest relatives Morelia macburniei and Morelia metcalfei, but failed to enumerate characters other than those cited above. Hence, the name cannot clearly be assigned to a specimen and this name is therefore considered a *nomen dubium* (APP5). Mense (2006), Henderson and Powell (2007), and Swan (2007) did not list this taxon.

#### Morelia nauta

Harvey et al., 2000

##### Synonyms:

Australiasis nauta (Harvey et al.) – Hoser 2004, 2009 (APP8, see introduction)

Morelia nauta Harvey et al. – Henderson and Powell 2007

##### Holotype:

UTA 44482.

##### Type locality:

Yamdena Island, Tanimbar Island Group, Maluku (=Moluccas), Indonesia.

#### Morelia oenpelliensis

(Gow, 1977)

##### Synonyms:

Nyctophilopython oenpelliensis (Gow)– Hoser 2000

Morelia oenpelliensis Gow – Henderson and Powell 2007

#### Morelia riversleighensis

(Smith & Plane, 1985) [extinct species]

##### Synonyms:

Montypythonoides riversleighensis – Smith and Plane 1985

Morelia spilota (Lacépède) – Kluge 1993

Morelia antiqua Smith & Plane, 1985 – Scanlon 2001

Morelia riversleighensis – Scanlon, 2001

##### Holotype:

QM F 12926 (=AR4058), incomplete right maxilla.

##### Type locality:

Henk’s Hollow Local Fauna, Tertiary System C, approximately 3.6 km southwest of Tedford’s (1967) Site B, Riversleigh, northwestern Queensland, Australia. Late Oligocene - early middle Miocene (Scanlon 2001).

##### Remarks:

Smith and Plane (1985) described the two extinct species *riversleighensis* and *antiquus* from Australia. Kluge (1993) synonymized *antiqua* (name amended for gender by Scanlon 1992) with *olivaceus* Gray due to the lack of autapomorphies and great overall similarity and *riversleighensis* with *spilota* Lacépède. Scanlon (2001) synonymized *antiqua* with *riversleighensis*.

#### Morelia spilota

(Lacépède, 1804)

##### Synonyms:

Morelia riversleighensis (Smith & Plane, 1985) – Kluge 1993 (part)

##### Remarks:

(Hoser (2000, 2004) listed several subspecies of Morelia spilota at specific rank, without comment. Since no new evidence is available, these authors continue to treat them all as subspecies. These authors also treat the taxon Morelia harrisoni described by Hoser (2000) as a subspecies of Morelia spilota (see comments there).

##### Morelia spilota spilota

(Lacépède, 1804)

##### Morelia spilota cheynei

Wells & Wellington, 1984

##### Morelia spilota harrisoni

Hoser, 2000 [subspecies inquirenda, APP7]

###### Holotype:

AMNH R-82433.

###### Type locality:

Port Moresby, Central Province, Papua New Guinea.

###### Remarks:

Hoser (2000: 24) described this taxon at specific rank but considered it “similar in most respects to the others in the genus Morelia” separating it from Morelia spilota cheynei, Morelia spilota variegata, and Morelia spilota mcdowelli “by distribution” (APP1, APP2), and further stating that specimens of this taxon “tend to have a lower average ventral and subcaudal scale count than Morelia cheynei, Morelia variegata and Morelia macdowelli, however the sample seen is too small to conclude if this trend is general” (Hoser 2000: 25). Hoser’s concept of this taxon comprises several populations throughout New Guinea. The author referred to Barker and Barker (1999) for further diagnostic characters. Barker and Barker identified several different and distinct populations from New Guinea, which Hoser (2000) placed within this catch-all taxon. For the “Port Moresby” population Barker and Barker (1999) stated that they “exhibit some characteristics of both *variegata* and *mcdowelli* (…).Most Port Moresby carpets have longitudinally expanded lateral pale blotches and bold facial stripes from the eye to the nasal scale, as do *mcdowelli* in northern Queensland. The patterns on the tops of the heads are similar to *variegata*”. For the “Irian Jaya” (now West Papuan or Papuan) population they stated that “[a]t 2 and 3 years of age, some are even as black and gold as Morelia spilota cheynei”, but did not provide further information on the “Trans-fly” (PNG) or the “Northern New Guinea” populations. Hoser (2000: 25) finally stated that “Morelia harrisoni can best be definitively separated from the other species of Morelia by DNA analysis” (APP2). To the authors’s best knowledge, no such analysis has been carried out. Since the diagnostic characters provided by Hoser (2000) and by Barker and Barker (1999) overlap with those for other taxa of the Morelia spilota complex, this taxon is likely to be confused with them. We consider this taxon a *subspecies inquirenda* (APP7). Henderson and Powell (2007) did not recognize this taxon. Mense (2006) discussed this taxon as a subspecies of Morelia spilota, and O’Shea (2007: 134) wrote: “Papuan Carpet Python Morelia spilota ssp. The status of all New Guinea Carpet Pythons is controversial (…). The New Guinea populations are fragmented and isolated, and their taxonomic status and relationships have yet to be determined with certainty”. Until further research has established otherwise, these authors treat this taxon as a subspecies of Morelia spilota, as proposed by Mense (2006) and Flagle and Stoops (2009).

##### Morelia spilota imbricata

LA Smith, 1981

###### Synonyms:

Morelia macburniei Hoser, 2004

##### Morelia spilota mcdowelli

Wells & Wellington, 1984

##### Morelia spilota metcalfei

Wells & Wellington, 1985

##### Morelia spilota variegata

Gray, 1842

###### Remarks:

Prior to (Wells and Wellington (1984, 1985), this name comprised all the taxa now recognized at subspecific rank, excluding the nominate subspecies and Morelia spilota imbricata, but including the New Guinean populations. Now Morelia spilota variegata is taxonomically restricted to Northern Australia (Kend 1997, Mense 2006).

#### Morelia tracyae

Harvey et al., 2000

##### Synonyms:

Australiasis tracyae (Harvey et al.) – Hoser 2004, 2009 (APP8, see introduction)

Morelia tracyae Harvey et al. – Henderson and Powell 2007

##### Holotype:

UTA 44473.

##### Type locality:

Tobelo, Halmahera, Maluku (=Moluccas), Indonesia.

#### Morelia viridis

(Schlegel, 1872)

##### Synonyms:

Chondropython viridis (Schlegel) – Hoser 2000 (part)

Chondropython viridis viridis (Schlegel) – Hoser 2004 (part)

Chondropython viridis shireenae Hoser, 2004

Morelia viridis (Schlegel) – Henderson and Powell 2007

##### Distribution:

see Wilson and Heinsohn (2007)

##### Remarks:

Rawlings and Donnellan (2003) found molecular evidence for cryptic diversity within Morelia viridis, resulting in two genetically distinct races. The type locality for Morelia viridis is Aru Island, which applies to the “southern lineage”, including Australian specimens (Rawlings and Donnellan 2003) (also see Chondropython azureus). However, Rawlings and Donnellan (2003: 42) noted that “(…) the east/west limits of the distribution of the two lineages may not necessarily be at the extreme ends of the central cordillera or the island”, and hence, there may be even more lineages present. Finally, due to the absence of molecular genetic data from the holotype, the type locality Aru Island cannot definitely be confirmed.

### Genus Python

Daudin, 1803

#### Synonyms:

Aspidoboa Sauvage, 1884 – Hoser 2004

Helionomus Gray – Hoser 2004 (incorrect subsequent spelling, APP4)

Shireenhoserus Hoser, 2004 (junior synonym of Enygrus Wagler)

#### Distribution:

Head (2005) reported remains of an indeterminate python from Miocene-age strata of the Siwalik Group of Pakistan. From the known distribution of extant species, this is likely to be a species of Python.

#### Remarks:

Hoser (2004) split this genus into several genera, e.g., Aspidoboa Sauvage (for *breitensteini*, *brongersmai*, and *curtus*), Broghammerus Hoser (for *reticulatus*), and Shireenhoserus Hoser (for *anchietae* and *regius*). However, Hoser (2004) overlooked Enygrus Wagler, 1830 (also see McDowell 1979: 9–10, 28), which makes Shireenhoserus a subjective junior synonym of Enygrus Wagler. He further intended to resurrect Heleionomus Gray, 1842 (for *sebae* and *natalensis*) but spelt the name as “Helionomus”. This constitutes an incorrect subsequent spelling (APP4), although the name Helionomus was already listed in Gray 1841 but is considered a *nomen nudum* (see remarks for Heleionomus). Only *molurus* and *bivittatus* would have remained within Python. Evidence from genetic studies reveal that with the exception of *reticulatus* and *timoriensis*, which were placed into Broghammerus (see comments there) by Rawlings et al. (2008), no further splitting of the clade Python is indicated. Furthermore, the phylogenetic relationships of several species (e.g., *regius* and *anchietae*, *molurus* and *bivittatus*, and *sebae* and *natalensis*) have not been fully resolved (e.g., Douglas et al. 2010: fig. 4-6). Other groups (e.g. the *curtus*-group *sensu lato*) are currently under study.

#### Python anchietae

Bocage, 1887

##### Synonyms:

Shireenhoserus anchietae (Bocage) – Hoser 2004 (junior synonym of Enygrus Wagler).

Python anchietae Bocage – Henderson and Powell 2007

#### Python bivittatus

(Kuhl, 1820)

##### Distribution:

See Greene et al. (2007), Snow et al. (2007), Pyron et al. (2008), and Barker and Barker (2009) for notes on introduced populations in Florida, USA. For distribution in Nepal, see O’Shea (1998), for distribution in Asia see Pauwels et al. (2003), (Barker and Barker (2008, 2010). Barker and Barker (2010) considered records of the occurrence of *bivittatus* in the Sichuan Province deviant due to complete isolation from the natural range of *bivittatus* and therefore excluded the province from the range of occurrence. Records from Sumatra and Borneo are believed to be incorrectly identified (Haile 1958, Groombridge and Luxmoore 1991).

##### Remarks:

Jacobs et al. (2009) considered this taxon a valid species. Evidence for this placement was already provided by O’Shea (1998, 2007) and Barker and Barker (2008) who pointed out that isolated populations of *bivittatus* do exist within the distributional range of *molurus* along the southern Nepalese border and in north-east India as reported from Assam by O’Shea (2007). Jacobs et al. (2009) primarily referred to Barker and Barker (2008) when stating that the isolated populations appear to exist not only sympatrically but syntopically with *molurus* but maintain their own integrity by avoiding interbreeding. However, O’Shea (pers. obs.) has observed the species inhabiting different habitats. Python molurus appears to occur in dry sandy woodland whereas *bivittatus* prefers riverine forests and flooded grasslands. O’Shea had not observed the two species occurring sympatrically or syntopically. Jacobs et al. (2009: 12) stated that de Rooij (1917) had assumed the type locality of Kuhl’s (1820) concept of *bivittatus,* which was based on unverified pictures by Seba, to be in Indochina rather than in the Sundaland and that the populations occurring between China and Java may be considered Python molurus sondaica (sic) Werner 1899. Nevertheless, according to Jacobson et al (2009), Mertens (1930) fixed the type locality to Java without the designation of a neotype, which has led to nomenclatural problems. Mertens (1930) as well as (Werner (1909, 1930) and Pope (1935) assumed that Schlegel (1837) rather than Kuhl (1820) had introduced the name *bivittatus*. According to Jacobs et al. (2009), Mertens (1930) was aware that Schlegel’s (1837) composite concept of Python bivittatus included several python taxa, namely those from India (Python molurus) and from Africa (Python sebae), respectively.

##### Python bivittatus bivittatus

(Kuhl, 1820)

##### Python bivittatus progschai

Jacobs et al., 2009 [subspecies inquirenda, APP7]

###### Holotype:

ZFMK 87481, subadult male from SW-Sulawesi.

###### Type locality:

Known only from the southwest of Sulawesi.

###### Remarks:

Jacobs et al. (2009) separated this subspecies from the nominate form by its generally smaller size (up to 240 cm in TL), up to 50% smaller egg size, and the smaller size of the neonates as well as by slightly different patterning and scale counts.

#### Python breitensteini

Steindachner, 1880

##### Synonyms:

Python breitensteini Steindachner – Keoghet al. 2001

Aspidoboa breitensteini (Steindachner) – Hoser 2004

Python breitensteini Steindachner – Henderson and Powell 2007

##### Remarks:

Elevated to specific rank by Keogh et al. (2001).

#### Python brongersmai

Stull, 1938

##### Synonyms:

Python brongersmai Stull – Keoghet al. 2001

Aspidoboa brongersmai (Stull) – Hoser 2004

Python brongersmai Stull – Henderson and Powell 2007

##### Remarks:

Elevated to specific rank by Keogh et al. (2001).

#### Python curtus

Schlegel, 1872

##### Synonyms:

Python curtus Schlegel – Keoghet al. 2001

Aspidoboa curtus (Schlegel) – Hoser 2004

Python curtus Schlegel – Henderson and Powell 2007

##### Remarks:

Elevated to specific rank by Keogh et al. (2001).

#### Python euboicus

Römer, 1870 [extinct species, considered nomen dubium by Rage 1984]

##### Synonyms:

Python Euboicus Römer, 1870

Heteropython euboicus (Römer) – de Rochebrune 1880

Heteropython euboicus (Römer) – Kuhn 1939, 1963

Python euboicus Römer – Rage 1984

##### Holotype:

Fragment of the trunk portion of the vertebral column (25 vertebrae and ribs), left dentary. No accession number. According to Szyndlar (1991) the holotype is probably lost.

##### Type locality:

Kimi (Euboea, Greece), early Miocene (MN ?3).

##### Remarks:

See Szyndlar (1991) and Szyndlar and Rage (2003: 67–68) for further information.

#### Python europaeus

Szyndlar & Rage, 2003 [extinct species]

##### Synonyms:

Python sp. – Rage 1982; Ivanov 2000, 2002

Python europaeus Szyndlar & Rage, 2003

##### Holotype:

MNHN, VCO 29. One trunk vertebra.

##### Type locality:

Vieux Collonges (=Mont Ceindre), France, early/middle Miocene (MN 4/5).

##### Remarks:

See Szyndlar and Rage (2003: 68–72), and Rage and Bailon (2005: 427–428) for further information.

#### Python molurus

(Linnaeus, 1758)

##### Python molurus molurus

(Linnaeus, 1758)

##### Python molurus pimbura

(Deraniyagala, 1945) [subspecies inquirenda, APP7]

###### Synonyms:

Python molurus molurus (Linnaeus) (part)

Python molurus molurus – Constable 1949

Python molurus pimbura – Deraniyagala 1955

Python molurus molurus – Stimson 1969

###### Distribution:

First reported from Nunavil (Thenmarachi), Jaffna Peninsula, Sri Lanka by Abyerami and Sivashanthini (2008).

###### Remarks:

Hoser (2004) resurrected this taxon from the synonymy of Python molurus molurus without providing reasons for this action. Deraniyagala (1945) separated the subspecies from Python molurus molurus based on lower subcaudal scale counts and the irregular shape of the lateral markings. Dorsal midbody scale rows and ventral scale counts overlap those of the nominate subspecies. Constable (1949: 124) did not follow this placement and synonymized this taxon with the nominate subspecies, which was followed by Stimson (1969). A second paper by Deraniyagala (1955: 6) provided a more detailed description of the subspecies. Therein, he stated that this taxon is also separated from the nominate form “in generally possessing three preoculars instead of two” or four as stated by Wall (1921: 47) for some Indian populations of the nominate form. There appears to be a range in preocular scale counts across India, from three in the northeast, to four in the north-center, and two in northwest (O’Shea pers. obs.) but this data, from only a few specimens, requires further verification. Contrary to his findings in 1945, Deraniyagala (1955) reports this taxon to have “more subcaudals” than the nominate form, obviously a typographic error according to the scale count data provided therein. It seems likely that subsequent workers overlooked this latter work, since neither Stimson (1969) nor McDiarmid et al. (1999) or Henderson and Powell (2007) cited it. Several subsequent workers accepted the placement to the synonymy of the nominate form, but no further studies have been conducted on the *molurus*-complex. However, besides the lower subcaudal scale counts and the higher number of preoculars, the pink surface of the head may also constitite a morphological difference. (Boulenger (1890, 1893) and MA Smith (1943) recorded two preoculars for Python molurus, while Wall (1921) records three preoculars for specimens from Ceylon. Since Sri Lanka is a known biodiversity hot spot with a high level of endemism, this allopatric population may represent a cryptic species. Because of the evidence provided by Deraniyagala (1955), these authors tentatively list this taxon as a valid subspecies and call for further research regarding its true status (APP7).

#### Python natalensis

A Smith, 1840

##### Synonyms:

Python natalensis A Smith – Broadley 1999

Helionomus natalensis (A Smith) – Hoser 2004 (*nomen nudum*, also see remarks on Python)

Python natalensis A Smith – Henderson and Powell 2007

##### Distribution:

Notes on the distribution of this species can be found in Alexander (2007).

##### Remarks:

McDiarmid et al. (1999) refer to A. Smith 1833. According to Branch and Bauer (2005), the name “Python Natalensis” already appeared in A. Smith (1833) as well as in A. Smith (1838) but without a description. The name appeared again in A. Smith (1840), but this time was accompanied by a plate. Gray (1842) also cites A. Smith (1840) as do Branch and Bauer (2005). Elevated to specific rank by Broadley (1999).

#### Python regius

(Shaw, 1802)

##### Synonyms:

Shireenhoserus regia (Shaw) – Hoser 2004 (junior synonym of Enygrus Wagler).

Python regius (Shaw) – Henderson and Powell 2007

##### Remarks:

For notes on the natural history and distribution of this species, see Barker and Barker (2006).

#### Python sardus

(Portis, 1901) [extinct species, nomen dubium]

##### Synonyms:

Paleopython sardus – Portis 1901

Paleryx sardus (Portis) – Kuhl 1963

?Python sardus (Portis) – Rage 1984

##### Holotype:

Articulated palatine and anterior pterygoid fragment (not traced).

##### Type locality:

Monte Albu (=Alba?)(Sardinia) Italy, middle Miocene (MN 6 or 7+8).

##### Remarks:

Szyndlar and Rage (2003: 72–73) considered this name a *nomen dubium* as it is indistinguishable from other (extinct) Python.

#### Python sebae

(Gmelin, 1788)

##### Synonyms:

Helionomus sebae (Gmelin) – Hoser 2004 (*nomen nudum*, also see remarks on Python)

Python sebae (Gmelin) – Henderson and Powell 2007

##### Remarks:

Elevated to specific rank by Broadley (1999).

### Genus Rawlingspython

Hoser, 2009 [unavailable name (APP8)]

#### Type species:

Liasis perthensis Stull, 1932

#### Remarks:

Hoser (2009) introduced this name as a monotypic subgenus of Antaresia Wells and Wellington 1984.

### Genus Shireenhoserus

Hoser, 2004 [subjective junior synonym of Python and subjective junior synonym of Enygrus Wagler]

#### Type species:

Python anchietae Bocage, 1887

#### Remarks:

Hoser (2004) established this genus for the smaller African taxa Python anchietae and Python regius. Hoser (2004) overlooked the older name Enygrus Wagler, 1830 (see remarks under Python), relegating Shireenhoserus as a junior synonym. Moreover, after relocation of the two Asian taxa *reticulatus* and *timoriensis* the genus Python now forms a clade, including Python regius. The phylogenetic relationship between Python regius and Python anchietae has not yet been examined and separation would result in non-monophyly. Hence, the recognition of this genus is unwarranted and it is assigned to the synonymy of Python.

## Discussion and Conclusion

In taxonomy, there have always been “lumpers” and “splitters”, but neither trend is helpful when taken to the extreme. “Splitters” could easily achieve monophyly by placing every single species in its own monotypic genus. Equally, lumping all taxa together into large unmanageable genera may obscure phylogenetic relationships and evolutionary diversity. Thus, a well-balanced “middle-ground” between “lumping” and “splitting” based on scientific evidence is the most desirable approach. In truth, Pythonidae is a relatively small family currently containing 40 extant species in nine genera, as defined here, yet it has been the subject of unprecedented attention by both professional and amateur taxonomists resulting in both papers that clarify and papers that confuse the phylogenetic relationships within the family. Whereas some subspecies may be recognized, erecting additional monotypic genera and creating subgenera within small genera is unwarranted and destabilizes taxonomy. Stable nomenclature, however, is most important for “unambiguous communication about biodiversity” and names must be clearly assignable to specimens to allow “unambiguous identifications” (Pyle and Michel 2008: 40). Since pythons are also highly desired by both the skin and pet trades an established and widely accepted taxonomy with associated nomenclature is essential if they are to be protected and conserved. Any unnecessary and unscientific deviations from a well-founded taxonomy can only serve to further threaten already vulnerable wild populations.

## Supplementary Material

XML Treatment for Antaresia

XML Treatment for Apodora

XML Treatment for Aspidites

XML Treatment for Aspidoboa

XML Treatment for Australiasis

XML Treatment for Austroliasis

XML Treatment for Bothrochilus

XML Treatment for Broghammerus

XML Treatment for Chondropython

XML Treatment for Heleionomus

XML Treatment for Helionomus

XML Treatment for Jackypython

XML Treatment for Katrinus

XML Treatment for Leiopython

XML Treatment for Lenhoserus

XML Treatment for Liasis

XML Treatment for Montypythonoides

XML Treatment for Morelia

XML Treatment for Python

XML Treatment for Rawlingspython

XML Treatment for Shireenhoserus

## Appendix 1

A list of valid taxa of pythons recognized in this study. Doubtful names (*nomina dubia*) are not included.

**Antaresia** Wells & Wellington, 1984

**Antaresia childreni** (Gray, 1842)

**Antaresia maculosa** (Peters, 1873)

**Antaresia perthensis** (Stull, 1932)

**Antaresia stimsoni**(LA Smith, 1985)

Antaresia stimsoni stimsoni (LA Smith, 1985)

Antaresia stimsoni orientalis (LA Smith, 1985)

**Apodora** Kluge, 1993

**Apodora papuana**(Peters & Doria, 1878)

**Aspidites** Peters, 1877

**Aspidites melanocephalus** (Krefft, 1864)

**Aspidites ramsayi** (Macleay, 1882)

**Bothrochilus** Fitzinger, 1843

**Bothrochilus boa** Fitzinger, 1843

**Broghammerus** Hoser, 2004 *fide* Rawlings et al. 2008

**Broghammerus reticulatus**(Schneider, 1801)

Broghammerus reticulatus reticulatus (Schneider, 1801)

Broghammerus reticulatus jampeanus (Auliya et al., 2002)

Broghammerus reticulatus saputrai (Auliya et al., 2002)

**Broghammerus timoriensis**(Peters, 1876)

**Leiopython** Hubrecht, 1879

**Leiopython albertisii** (Peters & Doria, 1878)

**Leiopython bennettorum** Hoser, 2000

**Leiopython biakensis** Schleip, 2008

**Leiopython fredparkeri** Schleip, 2008

**Leiopython hoserae** Hoser, 2000

**Leiopython huonensis** Schleip, 2008

**Liasis** Gray, 1842

**Liasis dubudingala** Scanlon and Mackness, 2002 [extinct species]

**Liasis fuscus** Peters, 1873

**Liasis mackloti** (Duméril and Bibron, 1844)

Liasis mackloti mackloti (Duméril and Bibron, 1844)

Liasis mackloti dunni Stull, 1932

Liasis mackloti savuensis (Brongersma, 1956)

**Liasis olivaceus** Gray, 1842

Liasis olivaceus olivaceus Gray, 1842

Liasis olivaceus barroni LA Smith, 1981

**Morelia** Gray, 1842

**Morelia azurea** (Meyer, 1874)

**Morelia amethistina** (Schneider, 1801)

**Morelia boeleni** (Brongersma, 1953)

**Morelia bredli** (Gow, 1981)

**Morelia carinata** (LA Smith, 1981)

**Morelia clastolepis** Harvey et al., 2000

**Morelia kinghorni**Stull, 1933

**Morelia nauta** Harvey et al., 2000

**Morelia oenpelliensis**(Gow, 1977)

**Morelia riversleighensis** (Smith and Plane, 1985) [extinct species]

**Morelia spilota**(Lacépède, 1804)

Morelia spilota spilota (Lacépède, 1804)

Morelia spilota cheynei Wells & Wellington, 1984

Morelia spilota harrisoni Hoser, 2000

Morelia spilota imbricata LASmith, 1981

Morelia spilota mcdowelli Wells & Wellington, 1984

Morelia spilota metcalfei Wells & Wellington, 1984

Morelia spilota variegata Gray, 1842

**Morelia tracyae** Harvey et al., 2000

**Morelia viridis**(Schlegel, 1872)

**Python** Daudin, 1803

**Python anchietae** Bocage, 1887

**Python bivittatus**(Kuhl, 1820)

Python bivittatus bivittatus (Kuhl, 1820)

Python bivittatus progschai Jacobs et al., 2009

**Python breitensteini** Steindachner, 1880

**Python brongersmai** Stull, 1938

**Python curtus** Schlegel, 1872

**Python europaeus** Szyndlar & Rage, 2003 [extinct species]

**Python molurus** (Linnaeus, 1758)

Python molurus molurus (Linnaeus, 1758)

Python molurus pimbura Deraniyagala, 1945

**Python natalensis** A Smith, 1840

**Python regius** (Shaw, 1802)

**Python sebae** (Gmelin, 1788)

## Appendix 2

### A. Key to the genera of the Pythonidae

**Table:**

1.	Visible sensory pits absent	Aspidites
–	Visible sensory pits present	**2**
2.	Rostral unpitted	**3**
–	Rostral pitted	**6**
3.	No visible black pigmentation between the scales	**4**
–	Black pigmentation visible between the scales	Apodora
4.	Number of loreals fewer than 3	**5**
–	Number of loreals more than 3	Antaresia
5.	Head color not black, head distinct from neck, two pairs of prefrontals	Liasis
–	Head color black, head bearly distinct from neck, one pair of prefrontals	Bothrochilus
6.	Body unpatterned	Leiopython
–	Body patterned	**7**
7.	Lateroposterior margin of nasal exposed, plane of ventral position of postorbital is directed anterolaterally, neck is markedly narrower than the head in adults	Morelia
–	Lateroposterior margin of nasal is covered by prefrontal, plane ventral position of postorbital is directed anteriorly, neck is slightly narrower than the head in adults	**8**
8.	Well defined square or triangular supralabial pits, infralabials less well developed and not set in a groove	Python
–	Less well defined diagonal supralabial pits, infralabials placed in a longitudinal groove and ventrally in a fold	Broghammerus

### B. Key to the species and subspecies of the genera of Pythonidae

**Antaresia**

**Table:**

1.	Body color pale yellowish-brown to dark purplish-brown	**2**
–	Head and neck color yellowish to reddish-brown	**3**
2.	Midbody scale rows 35 or fewer, ventrals fewer than 250, 34–45 subcaudals	Antaresia perthensis
–	Midbody scale rows 35 or more, ventrals more than 250, 38–57 subcaudals	Antaresia childreni
3.	Dorsal pattern of ragged-edged dark blotches	Antaresia maculosa
–	Dorsal pattern of smooth-edged blotches	**4**
4.	Ventrals 260–302	Antaresia stimsoni stimsoni
–	Ventrals 243–284	Antaresia stimsoni orientalis

**Aspidites**

**Table:**

1.	Head and neck color glossy black, numerous dark brown crossbands, ventrals more than 310	Aspiditesmelanocephalus
–	Head and neck color yellowish to reddish-brown, black markings above the eyes, ventrals fewer than 305	Aspidites ramsayi

**Apodora**

**Table:**

	Black skin pigmentation visible between head scales, rostral and (at least) second supralabial with shallow pits, prefrontals small or absent, ventrals 358–374, 83–88 subdaudals, low number of teeth on the maxilla	Apodora papuanus

**Bothrochilus**

**Table:**

	Uniform brownish-black head barely distinct from the head, orange color body ring pattern that fades with age, lack of rostral and supralabial pits, low number of dentary teeth	Bothrochilus boa

**Broghammerus**

**Table:**

1.	Iris color olive-golden, midbody scale rows fewer than 64, ventrals fewer than 290	Broghammerus timoriensis
–	Iris color bright yellow to golden-orange, midbody scale rows 64 or more, ventrals more than 290	**2**
2.	Ventrals more than 330	Broghammerus reticulatus saputrai
–	Ventrals fewer than 330	**3**
3.	Ventrals fewer than 304	Broghammerus reticulatus jampeanus
–	Ventral more than 304	Broghammerus reticulatus reticulatus

**Leiopython**

**Table:**

1.	Dorsal color dark gray or blackish-blue fading to white on the flanks	Leiopython hoserae
–	Dorsal color yellow to brownish-violet fading to yellowish on the flanks	**2**
2.	One pair of enlarged parietals	Leiopython huonensis
–	Two pairs of enlarged parietals	**3**
3.	Whitish postocular spot absent	Leiopython fredparkeri
–	Whitish postocular spot present	**4**
4.	Two prefrontals, two or more loreals present	Leiopython bennettorum
–	One prefrontal, one loreal present	**5**
5.	Subocular absent, three labials enter the orbit	Leiopython albertisii
–	Subocular present, only two labials enter the orbit	Leiopython biakensis

**Liasis**

**Table:**

1.	Body unpatterned	**2**
–	Body patterned	**4**
2.	Midbody scale rows fewer than 50, ventrals fewer than 300	Liasis fuscus
–	Midbody scale rows more than 50, ventrals more than 300	**3**
3.	Midbody scale rows 61–72, 355–377 ventrals	Liasis olivaceus olivaceus
–	Midbody scale rows 58–63, 374–411 ventrals	Liasis olivaceus barroni
4.	Eyes pale or white	Liasis mackloti savuensis
–	Eyes silvery or dark	**5**
5.	Chin and infralabials yellowish of color, brownish ground color, females larger than male	Liasis mackloti mackloti
–	Chin and infralabials of white color, grayish to reddish-brown ground color, males larger than females	Liasis mackloti dunni

**Morelia**

**Table:**

1.	Dorsal scales rough or keeled, large round frontal scale	Morelia carinata
–	Dorsal scales smooth, frontal of different shape partly fragmented	**2**
2.	Two or more enlarged well-defined pairs of parietals	**11**
–	Small granular or fragmented head scales	**3**
3.	Body ground color shiny green with unpatternd head	Morelia azurea, Morelia viridis
	(Note: Morelia azurea is a cryptic species, only distinguishable by genetic markers)
–	Body ground color pale cream; red or brown with head pattern	**4**
4.	Loreal scales fewer than 28	**5**
–	Loreal scales more than 28	Morelia bredli
5.	Body pattern of speckled appearance	Morelia spilota spilota
–	Body pattern of pale and dark elements	**6**
6.	Lack of partial structure in the posterior margin of the nasal scale	**7**
–	Presence of partial structure in the posterior margin of the nasal scale	**8**
7.	Nostril not in contact with the internasals	Morelia spilota imbricata
–	Nostril in contact with the internasals	Morelia spilota metcalfei
8.	Dorsal color dark	**9**
–	Dorsal color pale cream with diagonal pale bars and lighter pattern, head pattern smudgy appearance	Morelia spilota mcdowelli
9.	Body ground color dark brown or blackish	Morelia spilota cheynei
–	Body ground color shade of brown or reddish-brown	**10**
10.	Body pattern consists of 60–70 pale rings	Morelia spilota variegata
–	Body pattern with pale rings but connected by two lateral pale lines	Morelia spilota harrisoni
11.	Ventrals fewer than 400, subcaudals fewer than 125, infralabials fewer than 22, parietal scales not fragmented	**12**
–	Ventrals more than 400, subcaudals more than 155, infralabials more than 22, parietal scales fragmented	Morelia oenpelliensis
12.	Overall glossy blackish head and body color with white or yellowish bars in the labials	Morelia boeleni
–	Overall head and body color variable	**13**
13.	Neck bar pattern absent	**14**
–	Neck bar pattern present	**16**
14.	Postocular stripe absent	**15**
–	Postocular stripe present	Morelia nauta
15.	Suboculars absent, single supraocular	Morelia kinghorni
–	Suboculars present, 2–3 supraoculars	Morelia clastolepis
16.	Iris color golden, 0–2 interparietals	Morelia amethistina
–	Iris color red, 2–3 interparietals	Morelia tracyae

**Python**

**Table:**

1.	Small or fragmented head scales	**2**
–	Large, well-developed head scales	**4**
2.	Midbody scale rows fewer than 75, subcaudal scale counts fewer than 50	**3**
–	Midbody scale rows more than 75, subcaudal scale counts more than 60	Python natalensis
3.	Ventral scale counts fewer than 210, subcaudals fewer than 38	Python regius
–	Ventral scale counts more than 250, subcaudals more than 46	Python anchietae
4.	Ventral scale counts fewer than 200	**5**
–	Ventral scale counts more than 200	**7**
5.	Ventral scale counts fewer than 167	**6**
–	Ventral scale counts more than 167	Python brongersmai
6.	Anterior pair of parietals not in contact or are only weakly contacting	Python curtus
–	Anterior pair of parietals in broad contact at the medial structure	Python breitensteini
7.	Dorsal midbody scale rows fewer than 75	**8**
–	Dorsal midbody scale rows more than 75	Python sebae
8.	Suboculars absent	**9**
–	Suboculars present, separating the supralabials from the orbit	**10**
9.	Two preoculars present, subcaudals 66–70	Python molurus molurus
–	Three preoculars present, subcaudals 57–65	Python molurus pimbura
	(Additional diagnostic information: longitudinal pink marking above the eyes, fewer dark blotches that also invade the ventral scutes)
10.	Pale centered saddles, pale-centered brown blotches	Python bivittatus bivittatus
	(Additional diagnostic information: attains larger size up to 5m in length)
–	Prevalent saddles with pale margins, increased incidence of ocellic blotches (more molurus-like)	Python bivittatus progschai
	(Additional diagnostic information: does not exceed 2.5 m in total length)
